# Primary health care and family medicine in Sierra Leone

**DOI:** 10.4102/phcfm.v11i1.2051

**Published:** 2019-07-30

**Authors:** Colan Robinson

**Affiliations:** 1 Kings Sierra Leone Partnership, Freetown, Sierra Leone

**Keywords:** family medicine, postgraduate training, Sierra Leone

## Abstract

This article is a country profile of Sierra Leone describing the state of primary health care and family medicine in early 2019. Family medicine in Sierra Leone faces many challenges but recent changes in the location of the training programme encourage optimism that it may become better established within the next few years.

## Introduction

**FIGURE 1:**
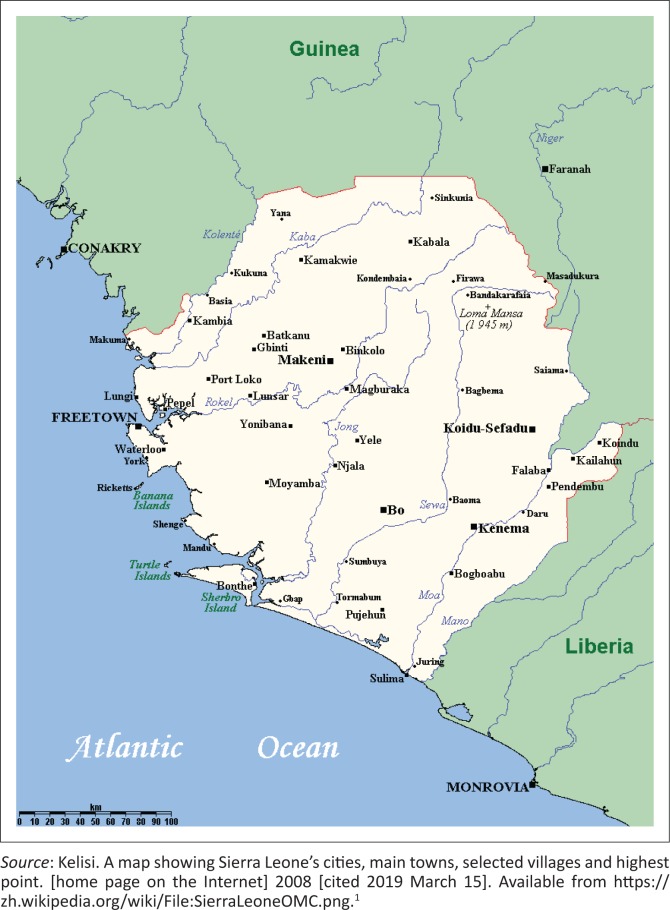
Map of Sierra Leone.

Sierra Leone is situated on the coast of West Africa with a population of 7.4 million, of which at least 1 million live in the urban environs of the capital Freetown (Figure 1).^1^ Its development has been hampered by a civil war between 1991 and 2002 and more recently by the Ebola outbreak of 2014, which claimed nearly 4000 lives, of whom over 90 were qualified health care workers (approximately 7% of the workforce).

### Ethical considerations

This article followed all ethical standards for research without direct contact with human or animal subjects.

## Burden of Disease and Description of Health System

The burden of disease is predominantly communicable disease, with 25% of deaths due to malaria, 9% due to hypertension and 9% due to maternal causes.^2^ In outpatients, the predominant conditions are tuberculosis, human immunodeficiency virus, hepatitis, hypertension and diabetes.

Ebola exacerbated an already challenging situation in terms of human resources. There are 1.4 doctors, nurses and midwives per 10 000 population compared to the most recent sustainable development goals threshold (set in 2016) of 44.5.^3^ In absolute terms, this equates to a qualified workforce of just over 1000 doctors, nurses and midwives, with an approximate shortage of 32 000. There is only one medical school in the country from which approximately 30 graduates pass out per year.

**FIGURE 2:**
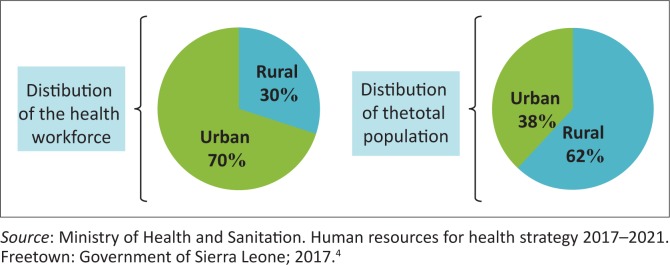
Urban versus rural distribution of health workforce versus population.

Within Sierra Leone, there is also an urban–rural split,^4^ with many doctors preferring to live and work in the urban areas (see Figure 2).^4^

With such a challenging combination of circumstances, it is not surprising that the health statistics for Sierra Leone are within the bottom quartile for least developed countries (LDCs). In its Health Sector Report,^3^ life expectancy is 51.3 years and under 5 mortality is between 120 and 156 per 1000 live births. Sierra Leone has the highest maternal mortality rate in the world at between 1165 and 1360 deaths per 100 000 live births compared to the average rate for LDC countries of 436 (all estimates by Demographic Health Survey [DHS] 2013 and United Nations [UN] 2015). This equates to a lifetime risk of dying from pregnancy of 1 in 21.

The Sierra Leone health care system is organised into two tiers of care: Peripheral Healthcare Units (PHUs) with an extended community health programme and secondary care which includes 21 district and three referral hospitals; there are also 45 private clinics and 27 private hospitals, mostly in the Freetown area.

The PHUs are further subdivided into maternal and child health posts serving a population of 500–5000, Community Health Posts serving a population of 5000–10 000 staffed by State-Enrolled Community Health Nurses (SECHNs) and Community Health Centres (CHCs) at chiefdom level serving a population of 10 000–30 000. Community Health Centres are run by Community Health Officers (CHOs) supported by SECHNs, lab assistants and environmental health workers. Community Health Officers have 3 years basic training. In total, there are approximately 1185 PHUs, of which 231 are CHCs.^4^ By late 2016, over 1000 CHOs had been trained since the grade was introduced in 1980.^5^ Recently, some CHOs have received further training in surgery and operative obstetrics to improve access to these services for rural populations.^5^

Free health care was introduced in 2010 for children under 5, pregnant or lactating women and following the devastating effects of the 2014 outbreak for Ebola survivors as well. The remainder of the population pay for their expenses; most patients will use traditional ‘country’ medicines as a first line and present late to the government health service. The affluent use private health care, either at home or abroad.

Postgraduate training in family medicine (FM) within Sierra Leone is accredited by the West African College of Physicians and owes a great deal to Drs Patrick Coker, Effie Gooding, Lynnette Palmer and Kojo Carew. Dr Coker set up postgraduate training in 2006 and Dr Palmer was the first Sierra Leonean trained consultant, followed by Dr Buck. Drs Palmer and Gooding helped establish FM as part of the undergraduate course. Until recently, postgraduate training was based in a private hospital, but in November 2018, it was moved to the government health service at Connaught Hospital in Freetown with the department based in the emergency centre – in keeping with the West African norm. This seems like a very positive move in that the government health service should benefit from the values and skills that FM brings to health care and FM will develop an academic base within a teaching institution, but it possibly comes at the cost of some loss of connection with the community health services. At present, Dr Adekunle (from Nigeria) is the head of department and there are four residents.

**FIGURE 3:**
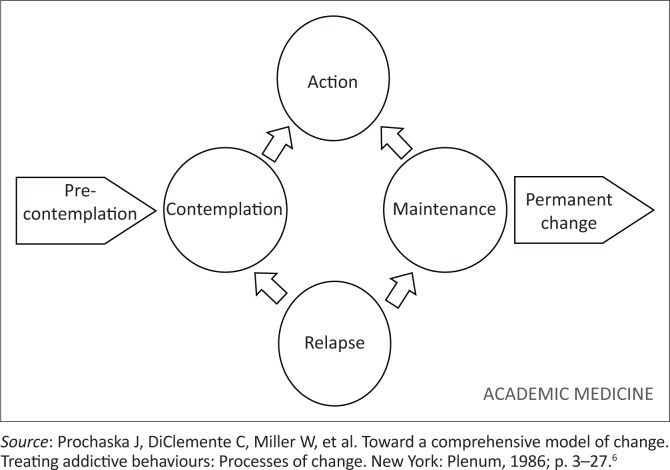
Stages of change model.

Referring to the ‘stages of change’ model (Figure 3)^6^ modified by Mash et al.,^7^ FM in Sierra Leone is at the action phase, in that there is an established heritage of postgraduate FM training here, it is timetabled in undergraduate course (2 weeks year 4, 3 weeks year 5 and 4 weeks year 6), and it is recognised by the professional council, but its place within the strategy of the Ministry of Health and Sanitation has not yet been formalised and remains more of an aspiration. The next cycle for an updated strategy begins in September 2021 and it would be important to establish FM within that strategy.

## Conclusion

FM is accepted as an entity within the medical community of Sierra Leone, but it faces challenges in terms of the available human resources, adjusting to its recent relocation within the structure of the main teaching hospital and the lack of co-ordination between the community health services and family physicians.
